# Knowledge and utilisation regarding untied fund among village level committees in selected villages of Wardha, Maharashtra

**DOI:** 10.1186/1753-6561-6-S1-P8

**Published:** 2012-01-16

**Authors:** Vikash R Keshri, AV Raut, AM Mehandale, BS Garg

**Affiliations:** 1 Mahatma Gandhi Institute of Medical Sciences, Wardha, India

## Introduction

Under the National Rural Health Mission (NRHM), village health and sanitation committees have been formed at village level in order to enable participation of people in monitoring and planning of health services. In Maharashtra state, considering the high prevalence of malnutrition, a nutrition component has been added and thus these committees are called village health nutrition and sanitation committees (VHNSC).

Government provides an untied fund of INR 10000 (USD 214.7) per year to VHNSC along with some broad guidelines on how to utilise the fund. To date, there is very little information available on knowledge about and utilisation of untied fund among VHNSC members.

Objective of this study was to assess the knowledge of VHNSC members about guidelines for utilisation of untied fund and to assess the knowledge of VHNSC members regarding actual utilisation of untied fund.

## Methods

We conducted a cross sectional study in 10 selected village across five health sub-centres in coverage area of the Anji primary health centre in Wardha district of Maharashtra. These villages are part of the field practice area of the Mahatma Gandhi Institute of Medical Sciences where authors are affiliated. We conducted the study during 15^th^ July to 15^th^ august 2010. We selected two villages from each of five health sub-centres; one village where health sub-centre was located, another from the coverage area of that health sub-centre selected using lottery method.

We administered a pre-tested questionnaire with 50% of VHNSC members in each selected village. Furthermore, we conducted in-depth interviews with the president and secretary of VHNSC. We analysed quantitative data using EpiInfo (version 6.04d). We analysed qualitative data manually using qualitative qualifier.

## Results

VHNSC members interviewed as part of this study had mean age of the 37.7 years and mean family income of INR 3700 (USD 79.5) per month. Male members constituted 61.9% of respondents. Majority of the respondents received education up to 9^th^ standard (57.2%) and had agriculture as the main occupation (54.7%).

Most (71.4%) of the VHNSC members were aware about NRHM and 85.7% members attended one or more VHNSC meeting. Nearly 61.4% said no meeting was held during last three months. Of those who respondents attended at least one VHNSC meeting, 85.7% were aware about untied fund and 77.7% were able to tell the correct amount of untied fund. Only 41.6% of respondents were aware of guidelines for utilisation of untied fund.

Regarding utilisation of fund during previous year, 77.7% of the respondents were unaware about where and how fund was utilised and 91.66% members reported that decision regarding utilisation of fund was made either by president or secretary of VHNSC without consulting the members.

Nearly 50% of VHNSC presidents and secretaries were aware of NRHM and some of them were aware about responsibilities of VHNSC. Majority of VHNSC presidents and secretaries were unaware about the guidelines for utilisation of untied fund. Most of the fund was utilised for supplementary diets to children attending *anganwadi* (supplementary nutrition centre).

## Discussion

Our study findings suggest that though untied fund is perceived by VHNSCs to be a good initiative, its utilisation is not as per the prescribed guidelines mainly due to limited awareness among VHNSC members on these guidelines. We suggest the need for training of VHNSC members and continuous monitoring of and support to VHNSC functioning in order to achieve its set objectives.

